# Diagnostic Accuracy and Turnaround Time of the Xpert MTB/RIF Assay in Routine Clinical Practice

**DOI:** 10.1371/journal.pone.0077456

**Published:** 2013-10-29

**Authors:** Nakwon Kwak, Sun Mi Choi, Jinwoo Lee, Young Sik Park, Chang-Hoon Lee, Sang-Min Lee, Chul-Gyu Yoo, Young Whan Kim, Sung Koo Han, Jae-Joon Yim

**Affiliations:** Division of Pulmonary and Critical Care Medicine, Department of Internal Medicine, Seoul National University College of Medicine, Seoul, Republic of Korea; Institute of Infectious Diseases and Molecular Medicine, South Africa

## Abstract

The Xpert MTB/RIF assay was introduced for timely and accurate detection of tuberculosis (TB). The aim of this study was to determine the diagnostic accuracy and turnaround time (TAT) of Xpert MTB/RIF assay in clinical practice in South Korea. We retrospectively reviewed the medical records of patients in whom Xpert MTB/RIF assay using sputum were requested. The sensitivity, specificity, positive predictive value (PPV) and negative predictive value (NPV) for the diagnosis of pulmonary tuberculosis (PTB) and detection of rifampicin resistance were calculated. In addition, TAT of Xpert MTB/RIF assay was compared with those of other tests. Total 681 patients in whom Xpert MTB/RIF assay was requested were included in the analysis. The sensitivity, specificity, PPV and NPV of Xpert MTB/RIF assay for diagnosis of PTB were 79.5% (124/156), 100.0% (505/505), 100.0% (124/124) and 94.0% (505/537), respectively. Those for the detection of rifampicin resistance were 57.1% (8/14), 100.0% (113/113), 100.0% (8/8) and 94.9% (113/119), respectively. The median TAT of Xpert MTB/RIF assay to the report of results and results confirmed by physicians in outpatient settings were 0 (0–1) and 6 (3–7) days, respectively. Median time to treatment after initial evaluation was 7 (4–9) days in patients with Xpert MTB/RIF assay, but was 21 (7–33.5) days in patients without Xpert MTB/RIF assay. Xpert MTB/RIF assay showed acceptable sensitivity and excellent specificity for the diagnosis of PTB and detection of rifampicin resistance in areas with intermediate TB burden. Additionally, the assay decreased time to the initiation of anti-TB drugs through shorter TAT.

## Introduction

Early and accurate detection of tuberculosis (TB) is important for the timely initiation of treatment and prevention of TB transmission. In addition, early detection of drug resistance is crucial for early treatment of multi-drug resistant (MDR) TB. Conventional methods for the diagnosis of TB have limitations in terms of early and accurate detection. Acid-fast bacilli (AFB) smears show short turnaround times (TAT) and high specificity [1], but lower and variable sensitivity [2], [3]. In South Korea, confirmation of mycobacterial culture and confirmation of conventional drug susceptibility tests (DST) takes approximately 43 days (19–77), and 91.5 days (51–170), respectively. [4].

The Xpert MTB/RIF assay, using RT-PCR for the TB specific *rpo*B gene, was validated for simple and rapid diagnosis of TB [5]. Detection of the *rpo*B gene is known to be specific for TB diagnosis, and mutation of the *rpo*B gene is associated with resistance to rifampicin (RIF). Using probes complementary to the *rpo*B gene, the Xpert MTB/RIF assay can identify M. TB and RIF resistance simultaneously in 2 h. In South Korea, the Xpert MTB/RIF assay was adopted for the diagnosis of TB since 2011.

Previous studies showed the high sensitivity and speed of the Xpert MTB/RIF assay for detection of TB and RIF resistance. When performed more than 3 times, the sensitivity of the Xpert MTB/RIF assay for detection of TB and RIF resistance was greater than 98% and 97%, respectively [5]. Time to detection of TB and RIF resistance could also be shortened, which led to earlier treatment [6]. However, these studies were performed in areas with a high burden of TB and in research settings. The aim of this study was to determine the diagnostic accuracy and TAT of the Xpert MTB/RIF assay in routine clinical practice in South Korea, a country with an intermediate TB burden, where the incidence of TB is 100 per 100,000 persons and 516 cases of MDR-TB were confirmed in 2011 [7].

## Methods

### Study Design

The design of this study was approved by the Institutional Review Board of Seoul National University Hospital. We retrospectively reviewed the medical records of patients in whom the Xpert MTB/RIF assay using sputum were requested due to suspicion of pulmonary TB between January 1 2011 and May 31 2013 at Seoul National University Hospital. Using data from these patients, we evaluated the accuracy of the Xpert MTB/RIF assay and compared the TAT of the assay with those of other TB tests. Obtaining consents from individual patients was waived by the Institutional Review Board.

### Accuracy of the Xpert MTB/RIF Assay for the Diagnosis of Pulmonary TB

We calculated the sensitivity, specificity, positive predictive value (PPV), and negative predictive value (NPV) of the Xpert MTB/RIF assay using sputum for the diagnosis of pulmonary TB. Diagnosis of pulmonary TB was confirmed by culturing M. TB from sputum for primary analysis. In this analysis, patients in whom M.TB was detected using the Xpert MTB/RIF assay, but M.TB isolation by mycobacterial culture failed, were excluded although all were treated with anti-TB drugs. We also calculated the sensitivity, specificity, PPV, and NPV of the Xpert MTB/RIF assay regarding the patients with positive Xpert MTB/RIF assay as having pulmonary TB.

### Accuracy of the Xpert MTB/RIF Assay for Detection of Rifampicin Resistance

We calculated the sensitivity, specificity, PPV, and NPV of the Xpert MTB/RIF assay using sputum to detect RIF resistance. Drug susceptibility tests (DST) using the absolute concentration method was used as a gold standard for detection of RIF resistance. Resistance to RIF was defined as ≥1% bacterial growth in Löwenstein–Jensen medium at a concentration of 40.0 µg/ml.

### Turnaround Time of the Xpert MTB/RIF Assay

The TAT of the Xpert MTB/RIF assay was compared with those of acid-fast bacilli (AFB) smears, cultures on liquid media or solid media, and conventional DST against anti-TB drugs. In addition, we compared the intervals from the request of diagnostic tests for TB to the initiation of anti-TB drugs between patient diagnosed as pulmonary TB using the Xpert MTB/RIF assay and pulmonary TB patients diagnosed without using the Xpert MTB/RIF assay. To perform this comparison, we matched twice as many TB patients diagnosed without using the Xpert MTB/RIF assay to patients diagnosed with Xpert MTB/RIF assay.

### Acid-fast Bacilli (AFB) Smear, Mycobacterial Culture, and the Xpert MTB/RIF Assay

Patients were asked to submit one spot and one or two subsequent morning sputa for AFB smear. One additional sputum was submitted for Xpert MTB/RIF assay. All sputum specimens were pretreated with equal volumes of 4% sodium hydroxide and centrifuged at 3000×*g* for 20 min. AFB smears were performed using Auramine-Rhodamine fluorescent staining and confirmed by Ziehl–Neelsen staining. Sediment was cultivated on Ogawa medium for 9 weeks in 5–10% CO_2_ incubators, as well as in BACTEC™ MGIT™ for 6 weeks. Once cultured, the isolation of M.TB was confirmed using the Gen-Probe® method (Gen-Probe, San Diego, CA, USA) [8].

The Xpert MTB/RIF assay was performed and interpreted according to the manufacturer’s instructions. Sputum specimens were lodged in the Xpert MTB/RIF assay cartridges, and tests were performed within 24 h after sputum submission.

### Statistical Analysis

The sensitivity, specificity, PPV and NPV of the Xpert MTB/RIF assay were calculated and 95% confidence intervals were estimated. Clinical data of included patients were described with the medians and ranges. Chi-square tests and Fisher’s exact tests were used for comparison of categorical variables, and independent *t*-tests, Mann-Whitney tests and Kruskal-Wallis tests were used to compare continuous variables. All analyses were performed using the SPSS software, version 19.0 (SPSS Inc., Chicago, IL, USA).

## Results

### Patients’ Characteristics

Between Jan 1 2011 and May 31 2013, the Xpert MTB/RIF assay using sputum was requested in 681 patients with suspicion of pulmonary TB. The median age of these patients was 61 years and 426 (62.5%) were male. Median number of submitted samples for each patients was 2 for AFB smear and mycobacterial culture. A total of 84 patients (12.3%) had diabetes. Among 681 pulmonary TB suspects, culture-proven pulmonary TB was diagnosed in 156 patients (22.9%). In addition, 59 patients were diagnosed with pulmonary TB based on their symptoms and radiographic findings, although M. TB was not cultured from their sputum (Table 1).

**Table 1:** Characteristics of the 681 patients in whom the Xpert MTB/RIF assay was requested.

Characteristics	*Number* (%)
Age, years, median (IQR‡)	61 (47.5–73.0)
Male	426 (62.5)
Number of submitted samples for AFB smear and mycobacterial culture, median (IQR)	2(1.0–3.0)
Co-morbidities	
Diabetes mellitus	84 (12.3)
Chronic kidney disease	44 (6.5)
Organ transplantation	23 (3.4)
HIV/AIDS	5 (0.7)
Malignancy	168 (24.7)
Final diagnosis	
Pulmonary tuberculosis	215 (31.6)
Bacteriologically confirmed	156 (22.9)
Clinically suggested	59 (8.7)
Bacterial pneumonia	123 (18.1)
Benign Pulmonary nodule(s)	46 (6.8)
Nontuberculous mycobacterial lung disease	31 (4.6)
Lung cancer	27 (4.0)
Bronchiectasis	27 (4.0)
Inactive TB sequelae	19 (2.8)
Extrapulmonary TB without pulmonary TB	23 (3.4)
Chronic bronchitis	11 (1.6)
Others*	161 (23.1)

‡ Interquartile range.

* Chronic obstructive pulmonary disease, 8; empyema, 6; asthma, 6; pulmonary thromboembolism, 4; *Pneumocystis jirovecii* pneumonia, 3; *bronchiolitis obliterans*, 2; *bronchiolitis obliterans* organizing pneumonia, 2; fungus ball, 2; postnasal drip, 2; interstitial lung disease, 2; radiation pneumonitis, 2; sarcoidosis. 2; sinusitis, 2; lung abscess, 1; pulmonary aspergillosis, 1; hypersensitivity pneumonitis, 1; gastroesophageal reflux disease, 1; pneumoconiosis, 1; being observed without definite diagnosis 113.

### Diagnostic Accuracy of the Xpert MTB/RIF Assay for Pulmonary TB

The sensitivity of the Xpert MTB/RIF assay for diagnosis of pulmonary TB was 79.5% (124/156) and specificity was 100.0% (505/505), while PPV was 100.0% (124/124) and NPV was 94.0% (505/537). Among patients with positive sputum AFB smears, sensitivity, specificity, PPV and NPV of the Xpert MTB/RIF assay was 88.9% (56/63), 100.0% (16/16), 100.0% (56/56), and 69.6% (16/23), respectively. Meanwhile, the sensitivity, specificity, PPV and NPV of the Xpert MTB/RIF assay were 73.1% (68/93), 100.0% (489/489), 100.0% (68/68), and 95.1% (489/514) among patients with negative sputum AFB smears. In the positive sputum AFB smear group, the sensitivity (*p* = 0.017) and PPV (*p*<0.001) were higher than in the negative sputum AFB smear group. (Table 2).

**Table 2:** Diagnostic accuracy of the Xpert MTB/RIF assay using sputum specimens for the diagnosis of pulmonary tuberculosis (Bacteriologically confirmed cases).

Detection of *M. tuberculosis*	Total patients (n = 661)	Patients with positive sputum AFB smears (n = 79)	Patients with negative sputum AFB smears (n = 582)
Sensitivity, % (95% CI†)	79.5(124/156) (72.1–85.4)	88.9 (56/63) (77.8–95.0)	73.1 (68/93) (62.8–81.5)
Specificity, % (95% CI)	100.0(505/505) (99.1–100.0)	100.0 (16/16) (75.9–100.0)	100.0 (489/489) (99.0–100.0)
Positive predictive value, % (95% CI)	100.0(124/124) (96.3–100.0)	100.0 (56/56) (92.0–100.0)	100.0 (68/68) (93.3–100.0)
Negative predictive value, % (95% CI)	94.0(505/537) (91.6–95.8)	69.6 (16/23) (47.0–85.9)	95.1 (489/514) (92.8–96.8)

† Confidence interval.

In 20 patients, the Xpert MTB/RIF assay with sputum was positive but *M. tuberculosis* was not cultured. Because the clinical features and radiologic findings of these patients were suggestive of pulmonary TB, these patients were treated with anti-TB drugs. These patients were excluded from the calculation of the sensitivity, specificity, positive predictive value, and negative predictive value.

The sensitivity of the Xpert MTB/RIF assay was 81.8% (144/176) for diagnosis of pulmonary TB if we classified not only patients with culture-proven TB but also patients with positive Xpert MTB/RIF assay as having pulmonary TB. (Table 3).

**Table 3:** Diagnostic accuracy of the Xpert MTB/RIF assay and mycobacterial culture using sputum specimens for the diagnosis of pulmonary tuberculosis.

	Xpert MTB/RIF assay (n = 681)	Mycobacterial culture (n = 681)	*p*-value
Sensitivity, % (95% CI)	81.8(144/176) (75.1–87.1)	88.6(156/176) (82.8–92.8)	0.071
Specificity, % (95% CI)	100.0(505/505) (99.1–100.0)	100.0 (505/505) (99.1–100.0)	
Positive predictive value, % (95% CI)	100.0(144/144) (96.8–100.0)	100.0 (156/156) (97.0–100.0)	
Negative predictive value, % (95% CI)	94.0(505/537) (91.6–95.8)	96.2 (505/525) (94.1–97.6)	0.105

In this analysis, bacteriologically confirmed cases as well as Xpert MTB/RIF assay positive cases were regarded as having pulmonary tuberculosis.

Among 79 patients with positive sputum AFB smears, the results of the Xpert MTB/RIF assay were negative in 23 patients. In 7 of these 23 patients, pulmonary TB was confirmed by culturing M.TB from their sputum. Nontuberculous mycobacteria (NTM) instead of M.TB were cultured from the sputum of the other 16 patients and these patients were excluded from the analysis (Table 2).

### Accuracy of the Xpert MTB/RIF Assay for Detecting Rifampicin Resistance

Conventional DST failed in 29 of 156 patients with culture-confirmed pulmonary TB. These 29 patients were excluded from the accuracy analysis using the Xpert MTB/RIF assay for the detection of RIF resistance. Using conventional DST, RIF resistance was identified in 14 of 127 patients with available DST results. The Xpert MTB/RIF assay correctly detected RIF resistance in 8 of 14 patients. Subsequently, the sensitivity of the Xpert MTB/RIF assay was 57.1%. False-positive RIF resistance using the Xpert MTB/RIF was not detected. Consequently, the specificity (113/113) and PPV(8/8) of the assay were 100%.

In five of six patients in whom the Xpert MTB/RIF assay failed to detect RIF resistance, the assay also failed to detect the presence of M.TB. If these five patients were excluded from the analysis, the sensitivity was 88.9% (8/9) and the specificity to 100% (90/90). In addition, PPV was 100% (8/8) and NPV was 98.9% (90/91) (Table 4).

**Table 4:** Diagnostic accuracy of the Xpert MTB/RIF assay using sputum specimens to detect rifampicin resistance among bacteriologically confirmed pulmonary tuberculosis in whom conventional DST results were available.

Rifampin resistance detection	Total TB patients with available results of conventional DST (n = 127)	Patients in whom pulmonary TB was confirmed by M. TB culture as well as the Xpert MTB/RIF assay (n = 99)
Sensitivity, % (95% CI)	57.1(8/14) (29.6–81.2)	88.9(8/9) (50.7–99.4)
Specificity, % (95% CI)	100.0(113/113) (95.9–100.0)	100.0(90/90) (94.9–100.0
Positive predictive value, % (95% CI)	100.0(8/8) (59.8–100.0)	100.0(8/8) (59.8–100.0)
Negative predictive value, % (95% CI)	94.9(113/119) (88.9–9.79)	98.9(90/91) (93.2–99.9)

### Turnaround Times (TAT) of the Xpert MTB/RIF Assay, AFB Smear, Mycobacterial Culture and Drug Susceptibility Tests

The median TAT from requested Xpert MTB/RIF assays to reported laboratory results was 0 days (0–1 days). This was significantly shorter than those of AFB smears (median 1 day, 0–1 days), culture on liquid (median 14 days, 10.25–17.75 days) or solid (median 24 days, 17–30 days) media, and drug susceptibility tests based on solid media (median 78 days, 65–96 days) (Table 5). Median TAT from requested Xpert MTB/RIF assays to confirmation of results by physicians was 6 days (3–7 days) in outpatient settings. This was also significantly shorter than other tests (Table 5).

**Table 5:** Turnaround time of the Xpert MTB/RIF, AFB smear, liquid/solid culture and drug susceptibility test.

Variables	Report of results from laboratory,days, median (IQR‡)	*p*-value*	Confirmation of results by dutyphysician, days, median (IQR)	*p*-value*
Xpert MTB/RIF assay	0 (0–1)	Ref.	6 (3–7)	Ref.
AFB smear	1 (0–1)	<0.001	12 (7.0–19.25)	0.001
Liquid culture	14 (10.25–17.75)	<0.001	21 (16.25–30.75)	<0.001
Solid culture	24 (17–30)	<0.001	38.5 (25.75–50.25)	<0.001
Drug susceptibility test	78 (65–96)	<0.001	90 (75.75–106.0)	<0.001

* *p*-values are from comparisons between tests and the Xpert MTB/RIF assay.

‡ Interquartile range.

Among pulmonary TB patients diagnosed using the Xpert MTB/RIF assay, anti-TB treatment was initiated median 7 days (4–9 days) after the evaluation for TB. However, among pulmonary TB patients in whom the Xpert MTB/RIF assay was not performed, the median time to initiation of anti-TB treatment was 21 days (7–33.5 days) (Table 6).

**Table 6:** Comparison of the clinical characteristics of patients diagnosed with pulmonary TB with or without the Xpert MTB/RIF.

Characteristics	Patient diagnosed TB using theXpert TB/RIF assay (n = 43) *n* (%)	Patient diagnosed TB not using theXpert TB/RIF assay (n = 86) *n* (%)	*p*-value
Age, years, median (IQR‡)	52 (32.0–70.0)	53 (39.75–67.50)	0.129
Male	22 (51.2)	44 (51.2)	1.000
Past history of treatment for pulmonary TB	10 (23.2)	12 (20.9)	0.464
Smoking status			0.636
Current	9 (17.3)	13 (15.1)	
Ex-smoker	7 (13.5)	18 (20.9)	
Never	17 (32.7)	40 (46.5)	
Co-morbidities			
Diabetes mellitus	8 (18.6)	12 (13.9)	0.491
Chronic kidney disease	1 (2.3)	4 (9.3)	0.664
Malignancy	8 (18.6)	10 (11.6)	0.281
Organ transplantation	0 (0)	2 (2.3)	0.552
Time to treatment after initial evaluations forpulmonary TB, days, median (IQR)	7 (4–9)	21 (7–33.5)	<0.001

‡ Interquartile range.

## Discussion

As an initial diagnostic method of M.TB, the accuracy and speed of the Xpert MTB/RIF assay have been demonstrated in previous studies. However, many of these were performed in low-income countries with limited medical resources [5], [6]. Our study was performed in tertiary referral hospitals in South Korea, where the annual incidence of TB was 100/100,000 on 2011 [7], and medical resources such as solid and liquid mycobacterial culture systems, as well as bronchoscopy or computed tomography, are readily available.

In our study, the sensitivity, specificity, PPV and NPV of the Xpert MTB/RIF assay for diagnosis of pulmonary TB were 79.5%, 100.0%, 100.0% and 94.0% respectively. The sensitivity in our study was lower than 90.4%, which was also reported in a recent meta-analysis [9]. Sensitivity of RIF resistance detection was 57.1%, but specificity and PPV were both 100.0%. The TAT of the Xpert MTB/RIF assay was shorter than AFB smears, mycobacterial culture and DST in terms of time to report of results, as well as time to confirmation of results by the physician. Consequently, the Xpert MTB/RIF assay shortened the time to initiation of anti-TB drugs by median 14 days.

The threshold of M.TB detection using the Xpert MTB/RIF assay is affected by the number of colonies in the sample [10]. Because smear-negative pulmonary TB reflects the lower burden of M.TB [11] and smear sensitivity correlates with quantitative growth [12], the sensitivity of the Xpert MTB/RIF assay could be lowered in smear-negative group. In previous studies of the Xpert MTB/RIF assay in high-TB burden countries [5], [6], [13], the proportion of smear-negative pulmonary TB was lower than that of smear- positive pulmonary TB. However, in South Korea, the proportion of sputum-negative pulmonary TB (61%) exceeded smear-positive TB (39%) [7]. In our study, 59.6% of culture confirmed pulmonary TB was sputum smear-negative. This could explain the relatively lower sensitivity (79.5%) of the Xpert MTB/RIF assay. However, we should take into account the fact that two or three sets of sputa samples were submitted for M.TB culture but the Xpert MTB/RIF assays were performed using only one sputum sample.

In our study, of 681 TB suspects, 156 patients were diagnosed with pulmonary TB with confirmation by mycobacterial culture. However, mycobacterial culture failed to identify M.TB in 59 patients who were concluded to have pulmonary TB based on the symptoms and radiographic findings. The fact that approximately one third of pulmonary TB cases could not be identified by mycobacterial culture suggests that more sensitive diagnostic methods are required. In fact, the Xpert MTB/RIF assay yielded positive results in 20 of these 59 TB suspects without bacteriological confirmation. This observation suggested that the Xpert MTB/RIF assay could be a complimentary test for the diagnosis of TB, including in the regions in which mycobacterial cultures are readily available.

In our study, the specificity and PPV of the Xpert MTB/RIF assay were each 100%. The high specificity and PPV of the assay are useful in countries such as South Korea, where the incidence of NTM is increasing rapidly [14]. Because of the considerable overlap in clinical characteristics between pulmonary TB and NTM lung diseases [15], exclusion of NTM lung diseases among patients with positive sputum AFB smears is crucial. Because the Xpert MTB/RIF assay showed high PPV of the Xpert MTB/RIF assay, physicians could exclude NTM lung disease and initiate anti-TB medication promptly among patients with both smear-positive and the Xpert MTB/RIF assay positive results.

The detection of MDR-TB is important because a prolonged treatment duration is required, but treatment success rates are low. Because RIF resistance serves as a surrogate of multidrug resistance [16], detection of RIF resistance using the Xpert MTB/RIF assay could be used to screen patients with MDR-TB. In our study, 14 patients were diagnosed with MDR-TB based on conventional DST, and 8 were shown to have RIF-resistance using the Xpert MTB/RIF assay. The low sensitivity of RIF-resistance detection was affected by the lower burden of M.TB, as discussed above. Meanwhile, in five MDR-TB patients, the Xpert MTB/RIF assay could not detect the presence of M.TB as well as RIF resistance. Thus, we must keep in mind that MDR-TB cannot be excluded based on the result of Xpert MTB/RIF assay when the assay shows negative results for M.TB.

Mathematically, the PPV of the Xpert MTB/RIF assay for RIF resistance varies according to the prevalence of MDR-TB. PPV for RIF-resistance is expected to be less than 70% based on the prevalence of MDR-TB in South Korea [17], [18]. Since the Xpert MTB/RIF assay can provide false positive results, selection of appropriate anti-TB drugs is difficult for patients with RIF-resistant TB based on the results of the Xpert MTB/RIF assay alone [19]. However, in our study, the specificity and PPV of the Xpert MTB/RIF assay were each 100% for RIF resistance. Although our observation suggests that false positive RIF-resistance detected by the Xpert MTB/RIF assay might be lower than expected, we should wait for the study including more patients with RIF-resistant TB before reaching a conclusion.

As expected, the TAT of the Xpert MTB/RIF assay in an outpatient setting was shorter than those of AFB smears, liquid culture, solid culture and DST in terms of interval to the report of results from the laboratory, as well as interval to the confirmation of results by physicians, in our study. In particular, the Xpert MTB/RIF assay shortened the time to initiation of anti-TB drugs by 14 days. However, the 5.5-day delay from the report of the Xpert MTB/RIF assays to confirmation by physicians suggested that the follow-up system requires improvement when the Xpert MTB/RIF assay is used in routine practice.

In conclusion, the Xpert MTB/RIF assay has acceptable sensitivity and excellent specificity for the diagnosis of pulmonary TB, as well as for the detection of RIF resistance in intermediate TB burden countries. In addition, the assay significantly shortened the time to anti-TB treatment.
